# Case Report: Add-on treatment with odevixibat in a new subtype of progressive familial intrahepatic cholestasis broadens the therapeutic horizon of genetic cholestasis

**DOI:** 10.3389/fped.2023.1061535

**Published:** 2023-02-14

**Authors:** Angela Pepe, Angelo Colucci, Martina Carucci, Lucia Nazzaro, Cristina Bucci, Giusy Ranucci, Angelo Di Giorgio, Pietro Vajro, Claudia Mandato

**Affiliations:** ^1^ Department of Medicine, Surgery and Dentistry “Scuola Medica Salernitana”, Pediatrics Section, University of Salerno, Baronissi (Salerno), Italy; ^2^ Pediatric Unit, University Hospital “San Giovanni di Dio e Ruggi d”Aragona”, Salerno, Italy; ^3^ Department of Gastroenterology, AORN Santobono- Pausilipon Children Hospital, Naples, Italy; ^4^ Pediatric Department, AORN Santobono- Pausilipon Children Hospital, Naples, Italy; ^5^ Department of Pediatric Gastroenterology Hepatology and Transplantation, Pediatric Hepatology, Gastroenterology and Transplantation, ASST Papa Giovanni XXIII, Bergamo, Italy

**Keywords:** PFIC, cholestasis, odevixibat, IBAT, itching

## Abstract

Odevixibat, an ileal bile acid transporter (IBAT) inhibitor, is effective for the treatment of pruritus in children diagnosed with progressive familial intrahepatic cholestasis (PFIC) type 1 and 2. There are no studies showing the efficacy of Odevixibat in children with different subtypes of PFIC. We describe the case of a 6-year-old girl with chronic cholestatic jaundice. In the last 12 months laboratory data showed high serum levels of bilirubin (total bilirubin x 2.5 ULN; direct bilirubin x 1.7 ULN) and bile acids (sBA x 70 ULN), elevated transaminases (x 3–4 ULN), and preserved synthetic liver function. Genetic testing showed homozygous mutation in ZFYVE19 gene, which is not included among the classic causative genes of PFIC and determined a new non-syndromic phenotype recently classified as PFIC9 (OMIM # 619849). Due to the persistent intensity of itching [score of 5 (very severe) at the Caregiver Global Impression of Severity (CaGIS)] and sleep disturbances not responsive to rifampicin and ursodeoxycholic acid (UDCA), Odevixibat treatment was started. After treatment with odevixibat we observed: (i) reduction in sBA from 458 to 71 μmol/L (absolute change from baseline: −387 μmol/L), (ii) reduction in CaGIS from 5 to 1, and (iii) resolution of sleep disturbances. The BMI z-score progressively increased from −0.98 to +0.56 after 3 months of treatment. No adverse drug events were recorded. Treatment with IBAT inhibitor was effective and safe in our patient suggesting that Odevixibat may be potentially considered for the treatment of cholestatic pruritus also in children with rare subtypes of PFIC. Further studies on a larger scale could lead to the increasing of patients eligible for this treatment.

## Introduction

Progressive familial intrahepatic cholestasis (PFIC) represents a heterogeneous group of rare genetic disorders caused by defects in bile secretion. Clinically, retention of the constituents of bile in blood mainly results in hypercholanemia with severe and hardly manageable pruritus typically appearing already in infancy or early childhood. Chronic cholestasis may slowly progress up to cirrhosis requiring liver transplantation (LT) during the pediatric age (1). PFIC can be classified into different types depending on the genetic defect in bile transporter. Three most prominent varieties are PFIC-1, 2 and 3, which are caused by mutations in ATP8B1, ABCB11, and ABCB4 genes, respectively. Even if the disease is suspected based on clinical and laboratory data, the diagnosis of certainty is carried out with genetic testing (1, 2). Odevixibat, a selective inhibitor of the ileal bile acid transporter (IBAT), is the first drug approved in the USA for the treatment of pruritus in PFIC patients aged ≥ 3 months and in Europe for the sole or an add-on treatment of PFIC patients aged ≥ 6 months. The drug is administered orally and acts in the intestine by binding reversibly to IBAT and reducing the reuptake of bile acids (BA) (3, 4). Odevixibat is effective for the treatment of cholestatic itching in PFIC1 and 2 (4); however, there is no experience in patients with a clinical phenotype suggestive for PFIC who don't have a classical mutation.

In the present follow-up study, we describe our experience on the use of Odevixibat for the treatment of severe cholestatic pruritus and sleep disturbances in a girl with a rare type of genetic cholestasis whose detailed case report was recently published (5).

## Case description

Briefly, a now 6-year-old girl born to consanguineous Moroccan parents was assessed at two months of life due to hepatosplenomegaly, high gamma-glutamyl transferase (GGT)/high serum BA cholestatic jaundice with preserved hepatocellular synthetic function. Biliary atresia was ruled out by intraoperative cholangiography. Several cholestasis etiologies were excluded with a Next Generation Sequencing (NGS) based liver panel which did not reveal genes mutations of either known PFICs/inborn errors of metabolism or structural cholestasis (e.g., ductal plate malformations/neonatal sclerosing cholangitis) (5) (Supplementary Table S1). At age of 4 years she underwent genetic WES (whole exome sequencing) showing hereditary sitosterolemia (OMIM #618666) and, in addition, a homozygous mutation of ZFYVE19 (zinc finger fyve-type containing 19)/ANCHR (abscission/no cut checkpoint regulator) gene (p.Arg223Ter) supporting the diagnosis of a recently described and still poorly defined type of genetic cholestasis [PFIC9 (OMIM #619849)] (6, 7). Both parents were heterozygous for the same mutation.

Immunofluorescence analysis of primary cilia on the patient's cultured skin fibroblasts revealed a ciliary phenotype mainly characterized by fragmented cilia and centrioles abnormalities (6).

She was treated with ursodeoxycholic Acid (UDCA, 25 mg/kg/day), rifampicin (5 mg/kg/day) and vitamin K (10 mg/week). During the follow-up, the child's clinical and laboratory picture remained quite stable. At the age of 5 years, she had normal values of serum albumin and international normal ratio (INR < 1.4) with persistently elevated liver tests: serum transaminases x 3–4 times the upper limit of normal (ULN), serum conjugated bilirubin x 2 times ULN, and extremely high serum BA (up to 603 μmol/L). Abdominal ultrasound showed hepatomegaly with hypertrophy of the caudate lobe and splenomegaly. Elastography was suggestive of moderate- severe fibrosis (11.1 kPa). Esophagogastroduodenoscopy revealed mild hypertensive gastropathy and esophageal varices (F1). No episodes of esophageal bleeding have ever occurred during clinical monitoring, and she has never presented ascites or signs of hepatic encephalopathy.

Parents were instructed to complete the Caregiver Global Impression of Severity (CaGIS), and Caregiver Global Impression of Change (CaGIC), evaluating pruritus with a typical 1 to 5 evaluation scale intensity (i.e., not present to very severe) and a 1 to 7 change (i.e., very much improved to very much worse), respectively (8). In spite of choleretic treatments, the child had very severe and persistent itching as reported by her parents at each visit [CaGIS score of 5 (very severe) and CaGIC score of 4 (no change), respectively].

As symptoms caused sleep disturbances, and limitations in daily activities ultimately resulting in a severely diminished quality of life, a surgical option (biliary diversion) was taken into consideration but discarded due to an already likely cirrhotic evolution. Therefore, at age 6 years, she was treated with odevixibat to give her a chance of a more effective, and possibly safe medical treatment. The drug was prescribed to compassionate use, after being approved by local ethical committee. During the follow-up, bilirubin, liver function and serum BA levels were continuously monitored (Table 1).

**Table 1:** Serum levels of liver enzymes, bilirubin and serum bile acids, and INR before and during odevixibat treatment.

Laboratory parameters	Last 12 months^a^	Baseline	Day 7	Day 20	Day 30	Day 45	Day 60	Day 90
Serum BA μmol/L (ULN 6)	415 ± 159	458	82	61	202	101	84	71
Total Bilirubin mg/dl (ULN 1.2)	2.96 ± 0.32	1.8	1.61	1.79	1.81	2.06	1.7	1.42
Direct bilirubin mg/dl (ULN 0.5)	0.84 ± 0.07	0.77	0.6	0.64	0.7	0.68	0.62	0.62
INR (ULN 1.2)	0.73 ± 0.48	1.05	1.14	1.21	1.15	1.09	1.27	1.24
AST U/L (ULN 41)	188 ± 23	149	155	166	158	175	120	129
ALT U/L (ULN 45)	157 ± 18	117	130	151	160	150	108	116
GGT U/L (ULN 50)	357 ± 77	265	229	320	228	221	256	291
RIFAMPICIN (5 mg/kg/day)	(+)	(+)	(+)	(+) → (−)	(−) → (+)	(+)	(+)	(+)
UDCA (25 mg/kg/day)	(+)	(+)	(+)	(+)	(+)	(+)	(+)	(+)
ODEVIXIBAT (40 *μ*g/kg/day)	(−)	(−)	(+)	(+)	(+)	(+)	(+)	(+)

ALT, alanine aminotransferase; AST, aspartate aminotransferase; BA, bile acids, INR, international normalized ratio, GGT, gamma-glutamyl transpeptidase, UDCA, ursodeoxycholic acid, ULN, Upper limit of normal. (+), ongoing therapy; (−), not ongoing therapy.

^a^ Mean ± standard deviation.

As soon as after one week a dramatic reduction of serum BA and pruritic symptoms was observed. For this reason, on day 21, rifampicin was discontinued with the view of reducing the therapeutic burden. Ten days after rifampicin discontinuation, blood tests showed a mild increase in BA levels and worsening of pruritus, and therefore rifampicin was restarted. This was followed by a progressive reduction in serum BA and improvement of pruritus at the same extent previously observed. During the follow up there were no further changes in co-medication: UDCA and Vitamins ADEK were given at the same dosage all through this time. Transaminases were substantially stable.

**Figure 1:**
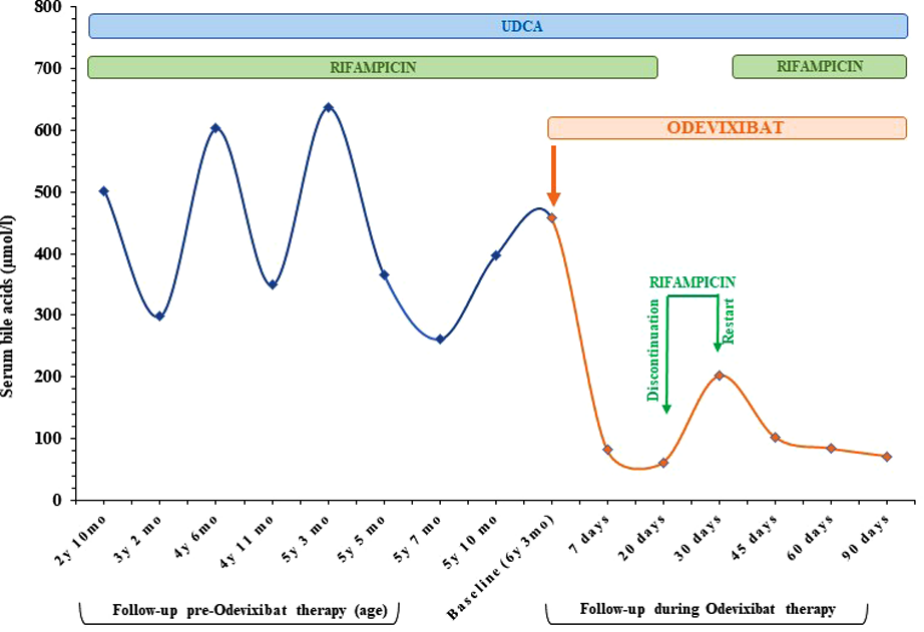
Serum bile acids in response to therapies. The start of Odevixibat therapy (orange arrow) coincides with a considerable reduction in serum bile acids compared to when the patient was treated only with UDCA (25 mg/kg/day) and rifampicin (5 mg/kg/day). mo, months; UDCA, ursodeoxycholic acid; Y, years.

At three months the absolute change of serum BA from baseline was of less 387 μmol/L (Figure 1). At the same time, the parents observed a substantial improvement of itching (CaGIC score of 1) whose intensity almost disappeared (CaGIS reduced from 5 to 1). Sleep disturbances were not anymore complained, and appetite improved. The child's nutritional status also improved: BMI z-score increased progressively from −0.98 to +0.56 after 3 months of therapy. No adverse drug events (ADEs) were recorded.

## Discussion

PFIC is a group of genetic disorders caused by defective bile acids transport which can lead to liver cirrhosis and end-stage liver disease. Pruritus, the most common manifestation that may dramatically worsen the quality of life, requires treatment with a little choice of (unfortunately often ineffective) drugs (7) and/or biliary diversion to interrupt enterohepatic circulation of bile acids. In the most severe and refractory cases, itching can be an indication for liver transplant. Odevixibat, an ileal apical sodium-dependent IBAT inhibitor, has been recently approved for treatment of pruritus in PFIC patients older than 3 months as the sole or an add-on drug with mild side effects. PEDFIC1 is the main phase 3 randomized controlled trial providing clinical evidence of Odevixibat efficacy and safety in patients with a clinical diagnosis of PFIC1 or PFIC2 (4).

Reports describing patients with rare subtypes of PFIC treated with Odevixibat are scanty. Slavetinsky et al., described the case of a 15-month-old male patient affected by PFIC2 with severe cholestatic pruritus enrolled in a clinical trial of Odevixibat. After the trial ended the child had to undergo partial external biliary diversion due to recurrence of symptoms previously well controlled by the drug. Odevixibat and partial external biliary diversion showed equal improvement of cholestasis (8). As seen with biliary diversion, Odevixibat seems also to improve hepatic fibrosis (4).

In the case here described, WES found a homozygous mutation of ZFYVE19/ANCHR (5), a gene somehow involved in both cilia function (5, 6, 9–17) and bile acid transport (18). Briefly, ZFYVE19 protein product mediates communication between endosomal sorting complexes for transport-III (ESCRT-III) machinery proteins and the vacuolar protein sorting 4 (VPS4) which are localized at the ciliary transition zone where they control centrosome duplication and template ciliogenesis as well. Dysregulation of this process may therefore impact complex patterns formation as seen also in other ciliopathies involving the liver (11–15, 19–21). Additionally, the ESCRT-III machinery molecules have been shown to be essential also for polarized trafficking of the apical membrane protein bile salt export pump (BSEP). As BSEP mediates the rate-limiting step of bile salt transport from hepatocytes into bile canaliculi, dysregulated ESCRT functions will result in subapical BSEP retention and cholestasis (18).

The choice of treating the patient only on the basis of the clinical phenotype of congenital intrahepatic cholestasis was challenging, however the brilliant clinical response was stunning since her quality of life and that of her family have dramatically improved. We believe that odevixibat may give the opportunity to treat children with intrahepatic cholestasis, even before identifying one of the increasingly reported spectrum of genetic mutations (9). Unfortunately, we cannot predict if the treatment will prolong the native liver survive, due to the still short follow-up.

The treatment was started at a minimum dose (40 µg/kg/day) and already after a week of treatment a reduction in serum BA of about 80% was observed. Adherence to therapy in pediatric population is favored by the fact that it can be taken in capsules or opened and sprinkled onto food. The patient did not present any of the most common adverse events previously described (vomiting, diarrhea, abdominal pain, serum aminotransferase levels elevations) (4).

It is interesting to note that, the discontinuation of rifampicin after 20 days of concomitant treatment with odevixibat resulted in a mild rise in serum BA and a resumption of mild itch. Therefore, rifampicin therapy was restarted and a clear-cut decreasing trend in serum BA was again observed. It can be supposed that, given the particularly high level of serum BA, the synergistic action of the two drugs could be essential to effectively reduce BA levels: rifampicin increases the metabolism of pruritogenic substances inducing cytochrome P-450 isoenzyme system and odevixibat eliminates pruritogens from the enterohepatic cycle (7).

## Conclusion

In conclusion, an odevixibat 3-month add-on treatment was effective, safe and well tolerated in our patient. Due to the success preliminarily reported also in other types of infantile cholestasis (e.g., Alagille syndrome) (3), we believe that our case may represent a milestone of treatment of clinical intrahepatic cholestasis in children as it gives new opportunity in improving quality of life of patients even before the genetic confirmation is reached. However, longer observation times and further studies are required to confirm the safety and effectiveness of this new therapeutic choice over the time.

## Data Availability

The original contributions presented in the study are included in the article/**Supplementary Material**, further inquiries can be directed to the corresponding author/s.
