# Supplementary material for: Case Report: Add-on treatment with odevixibat in a new subtype of progressive familial intrahepatic cholestasis broadens the therapeutic horizon of genetic cholestasis
Source: Front Pediatr. 2023 Feb 14;11:1061535. doi: 10.3389/fped.2023.1061535 (PMC9974160; doi:10.3389/fped.2023.1061535)
Supplement: Supplementary file 1 [file Table1.docx]

| **Table S1- Chronic cholestasis-related genes analyzed before performing WES** | |
| --- | --- |
| PFIC GENES | **OTHER LIVER PANEL GENES** |
| ATP8B1 | ABCB4, ACADM, AKR1D1, ALDOB, AMACR, ATP7B, BAAT, CYP7B1, *DCDC2,* DGUOK, EPHX1, FCYT, PKHD1, GAA, GALT, GBA, HSD3B7, JAG1, NOTCH2, LIPA, NPC1, NPC2, POLG, SERPINA1, SLC25A13, SMPD1, UGT1A1 |
| ABCB11 |  |
| ABCB4 |  |
| TJP2 |  |
